# Two-step lifting method using the Wang Intestinal Strap for laparoscopic radical resection of mid-low rectal cancer (with video)

**DOI:** 10.1093/gastro/goac041

**Published:** 2022-08-17

**Authors:** Yu Zeng, Feng Peng, Xiaosong Gong, Jianmei Yi, Chuangkun Li, Qing Wang, Jin Wang

**Affiliations:** Department of General Surgery, Zhuzhou Central Hospital, Zhuzhou, Hunan, P. R. China; Department of General Surgery, Zhuzhou Central Hospital, Zhuzhou, Hunan, P. R. China; Department of General Surgery, Zhuzhou Central Hospital, Zhuzhou, Hunan, P. R. China; Department of General Surgery, Zhuzhou Central Hospital, Zhuzhou, Hunan, P. R. China; Department of General Surgery, Zhuzhou Central Hospital, Zhuzhou, Hunan, P. R. China; Department of General Surgery, Zhuzhou Central Hospital, Zhuzhou, Hunan, P. R. China; Department of General Surgery, Zhuzhou Central Hospital, Zhuzhou, Hunan, P. R. China

## Introduction

Laparoscopic total mesorectal excision for mid-low rectal cancer is associated with several fundamental problems. A narrow pelvis, deep tumor location, obesity, and comparatively larger tumors lead to difficulties in performing clear dissection, accurately determining a safe distance from the lower margin of the tumor, and performing adequate lavage of the pelvic cavity distal to the tumor before sealing the rectum [1, 2]. In laparoscopy-assisted radical resection of mid-low rectal cancer, gauze and bulldog clamps are mainly used to bind and pull the intestine and its mesorectum and block the distal bowel [3]. Use of a bulldog clamp to block the intestines often fails when the bulldog clamp loosens and falls off. Gauze is widely used in clinical practice. However, it is relatively difficult to tie the intestine with gauze strips. Additionally, because of the lack of a self-locking function, gauze cannot completely block the intestine.

**Figure 1.**
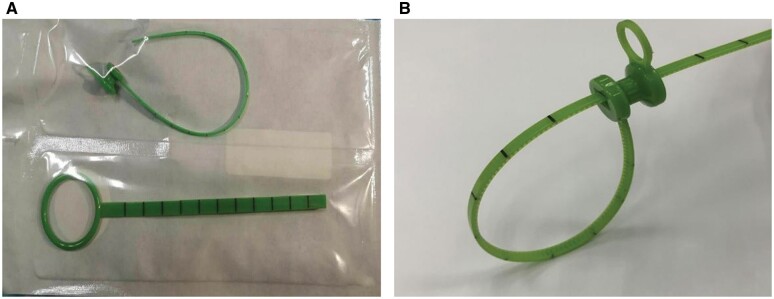
A single-use intestinal strapping and lifting device called the Wang Intestinal Strap. (A) The whole tool. (B) The locking section.

Therefore, we designed and developed the “disposable tubular viscera strapping and lifting tool” (patent number ZL2016.1.03866607) and its improved product, a single-use intestinal strapping and lifting device called the Wang Intestinal Strap (Figure 1). We applied the two-step lifting method to laparoscopic low anterior resection of mid-low rectal cancer and obtained satisfactory preliminary results.

## Surgical approach

The specific steps of the two-step lifting method during laparoscopic low anterior resection are as follows.

**Figure 2.**
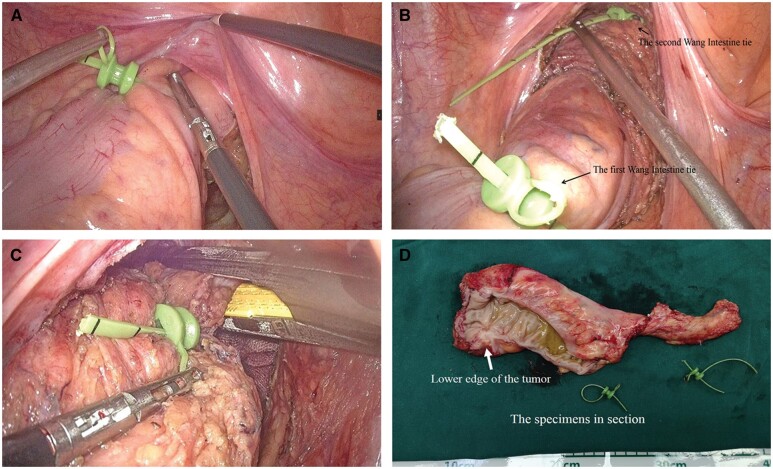
The two-step lifting method during laparoscopic low anterior resection of mid-low rectal cancer. (A) The first Wang Intestinal Strap is placed 2–3 cm above the peritoneal reflection. (B) The second Wang Intestinal Strap is placed after the distal edge of the tumor is confirmed. (C) The rectum is cut 1–2 cm away from the second Wang Intestinal Strap. (D) Surgical specimens.

Step 1: After the root dissection of the submesenteric vessels and mesenteric dissociation, the upper part of the rectum is dissociated to 2–3 cm above the peritoneal reflection and the first Wang Intestinal Strap is placed (Figure 2A). To maintain good traction force, clearly expose the space around the rectum and carry out accurate dissociation; the rectum and its mesorectum are strapped and pulled using the first Wang Intestinal Strap until the anatomic planes exceed the lower edge of the tumor and reach 2–3 cm above the dentate line.

Step 2: The second Wang Intestinal Strap is strapped to the rectum and its mesorectum to properly seal the intestinal cavity 1 cm below the lower edge of the tumor. The operative field is then well exposed and the distal rectal dissociation becomes easier to perform. Because the second Wang Intestinal Strap encloses the tumor entirely in the pre-resected intestine, thorough lavage through the anus can be performed to achieve a tumor-free and sterile effect (Figure 2B). Meanwhile, the second Wang Intestinal Strap marks the position of the distal end of the tumor. By pulling the second Wang Intestinal Strap, an Endopath stapler and endoscopic linear cutter can be conveniently used to cut the rectum at 1–2 cm from its distal end. In this way, a safe distance to the distal resection margin can be ensured (Figure 2C). More operative details are show in the Supplementary video.

## Practical application

Between January 2018 and October 2019, we performed laparoscopic-assisted anterior resection using a Wang Intestinal Strap in 37 patients with mid-low rectal cancer, while at the same time 32 patients underwent the same operation with gauze in our hospital. The use of the Wang Intestinal Strap reduced the difficulty of the operation, shortened the mean operative time (106 ± 16 vs 140 ± 28 min, *P* < 0.001), and decreased the mean intraoperative blood loss (82 ± 47 vs 130 ± 58 ml, *P* < 0.001). Moreover, the operations using the Wang Intestinal Strap facilitated proper sealing of the intestinal cavity and performance of tumor-free lavage, fitting the principles of radical tumor surgery and guaranteeing a safe distal resection margin of the tumor (2.7 ± 0.7 vs 2.2 ± 0.6 cm, *P* < 0.001).

## Discussion

The two-step lifting method using the Wang Intestinal Strap has the following advantages. First, the device is small and made of elastic materials, allowing it to be flexibly operated within a small space. This is beneficial in overcoming the operative difficulties caused by a narrow pelvis. Second, the elastic material and practical design of the Wang Intestinal Strap reduce the risk of tissue cutting and damage during the strapping and pulling process. Third, the device can pull the rectum and its mesorectum together forcefully and maintain traction continuously, thus effectively exposing adequate space around the rectum. Its practical value in this respect is highlighted especially in patients with obesity, huge tumors, and a narrow pelvis. Fourth, the Wang Intestinal Strap enables surgeons to seal the intestinal cavity distal to the tumor and conduct thorough lavage to achieve a tumor-free and sterile effect. Additionally, the position of the distal tumor resection edge can be marked with this instrument, providing convenience when using an Endopath stapler and endoscopic linear cutter with reloads to accurately measure the safe distance of the tumor’s distal resection margin. Finally, the tool significantly improves the success rate of sphincter preservation in patients with mid-low rectal cancer.

## Supplementary data

Supplementary data is available at *Gastroenterology Report* online.

## Funding

This work was supported by the Hunan Province Clinical Medical Technology Innovation Guidance Project (grant number 2020sk53903).

## Supplementary Material

goac041_Supplementary_Data

## References

[1] Chau J , Solomon J, Liberman AS et al Pelvic dimensions on preoperative imaging can identify poor-quality resections after laparoscopic low anterior resection for mid- and low rectal cancer. Surg Endosc 2020;34:4609–15. 31620910 10.1007/s00464-019-07209-8

[2] Kang L , Zeng Z, Luo S et al Transanal vs laparoscopic total mesorectal excision for rectal cancer: a multicenter randomized phase III clinical trial (TaLaR trial) protocol. Gastroenterol Rep (Oxf) 2021;9:71–6. 33747528 10.1093/gastro/goaa083 PMC7962745

[3] Holman FA , van der Pant N, de Hingh IH et al Development and clinical implementation of a hemostatic balloon device for rectal cancer surgery. Surg Innov 2014;21:297–302. 24172167 10.1177/1553350613507145

